# Enhancing basal cell carcinoma classification in preoperative biopsies via transfer learning with weakly supervised graph transformers

**DOI:** 10.1186/s12880-025-01710-4

**Published:** 2025-05-16

**Authors:** Johan Björkman, Sigrid Lagerroth, Jan Siarov, Filmon Yacob, Noora Neittaanmäki

**Affiliations:** 1 Department of Physics, Chalmers University of Technology, Gothenburg, Sweden; 2 Department of Laboratory Medicine, Institute of Biomedicine, Sahlgrenska Academy, University of Gothenburg, Gothenburg, Sweden; 3 Region Västra Götaland, Department of Clinical Pathology and Cytology, Sahlgrenska University Hospital, Gula Stråket 8, Gothenburg, 41345 Sweden; 4 Ekkono Solutions, Varberg, Sweden

**Keywords:** Basal cell carcinoma, Digital pathology, Deep learning, Weakly supervised, Graph transformer, Graph convolutional network, Transfer learning

## Abstract

**Background:**

Basal cell carcinoma (BCC) is the most common skin cancer, placing a significant burden on healthcare systems globally. Developing high-precision automated diagnostics requires large annotated datasets, which are costly and difficult to obtain. This study aimed to fine-tune a weakly supervised machine learning model to classify BCC in preoperative punch biopsies using transfer learning. By addressing challenges of scalability and variability, this approach seeks to enhance generalizability and diagnostic accuracy.

**Methods:**

The Basal Cell Classification (BCCC) dataset included 514 WSIs of punch biopsies (261 with BCC and 253 tumor-free slides), divided into training (70%), validation (15%), and test sets (15%). WSIs were split into patches, and features were extracted using a pretrained simCLR model trained on 1,435 WSIs from BCC excisions. Features were formed into graphs for spatial information and the processed by a Vision Transformer. Testing included finetuned and non-finetuned pre-trained models as well as a model trained from the scratch, evaluated on 78 WSIs from the BCCC dataset. The COBRA dataset of 3,588 WSIs (1,794 with BCC and 1,794 without) was used for external validation. Models classified no-tumor vs. tumor (two classes), no-tumor vs. low-risk vs. high-risk tumors (three classes), and no-tumor vs. four BCC subtypes (five classes).

**Results:**

The fine-tuned model significantly outperformed the non-fine-tuned pretrained model and the model trained from the scratch with accuracies of 91.7%, 82.1%, and 75.3% and with AUCs of 0.98, 0.95–0.98, and 0.91–0.97 for two, three, and five-class classification. On the external validation, accuracies were 84.9% and 70.5%, with AUCs of 0.92 and 0.89–0.91 for two and three-class classification, respectively. The ablation study revealed that the fine-tuned model outperformed the model trained from scratch, improving mean accuracy by 10.6%, 11.7%, and 13.1% on the BCCC dataset, as well as by 29.6% and 19.2% on the COBRA dataset.

**Conclusions:**

The results suggest that transfer learning not only enhances model performance on small datasets but also supports robust feature extraction in complex histopathology tasks. These findings reinforce the utility of pre-trained models in computational pathology, where access to large, labeled datasets is often limited, and task-specific challenges require nuanced understanding of the visual data.

**Supplementary Information:**

The online version contains supplementary material available at 10.1186/s12880-025-01710-4.

## Background

Basal cell carcinoma (BCC) is the most common form of cancer among the Caucasian population [1–2]. Although BCC rarely metastasizes and has an exceptionally low mortality rate [3], it can cause considerable morbidity since the tumor can grow aggressively causing local destruction of important anatomic structures [4]. An accurate classification is crucial for successful treatment [5].

The gold standard of diagnosis is histopathological evaluation. A punch biopsy is a common method for initial diagnosis of suspected lesions and often used in order to plan the final non-surgical or surgical treatment method [5]. For classification, an internationally recognized system from the World Health Organization divides BCC into either low-risk or high-risk, where risk refers to risk of recurrence [6]. The Swedish classification system (a.k.a. “Sabbatsberg’s model”) groups BCCs into four subtypes based on their aggressiveness based on growth patterns: nodular (type Ia), superficial (type Ib), medium-aggressive (type II) and high-aggressive (type III) [7]. Over 40% of BCC show mixed histology which can cause difficulties when assessing biopsies [8]. Consequently, there are significant discrepancies in the assessment of tumor subtypes as well as practices in reporting subtypes among pathologists [9–10]. Furthermore, the high and increasing BCC incidence rates cause significant increases in the workload for pathology laboratories and increasing health care costs [1, 11]. This combined with the global shortage of pathologists, necessitates the exploration of new solutions to speed up and simplify diagnosis [12].

In recent years digitalization has enabled the incorporation of machine learning (ML) to histopathological images. Ever since April 2017, when Philips IntelliSite digital scanner received FDA approval for use in digital pathology, the research on what is known as computational pathology has increased and is now witnessing what could be called a paradigm shift [12–13].

In supervised learning, the ML model is typically trained on labeled data, where labels can be applied at the image level, meaning each whole image is assigned a single label. This is often sufficient for classification tasks, as the model learns to differentiate between classes based on entire image features. However, in segmentation tasks or when finer granularity is required, pixel-wise annotations may be necessary. For instance, a pathologist labels specific areas of a whole slide image, such as regions containing tumor. This method relies heavily on the quality and specificity of the annotations, which will in turn have a significant effect on the performance of the trained models. The further drawback of this method is that it is difficult to collect substantial volumes of data as the process of labeling requires considerable time and effort. This limitation affects both the scalability and the diversity and representativeness of the dataset. The required laborious pixel-wise annotations by pathologists is time-consuming and limits the rapid development of this field due to lack of representative annotated datasets [14]. It has become apparent that less laborious methods are warranted.

To reduce the time-consuming tasks associated with supervised computational pathology, researchers have increasingly turned to weakly or unsupervised supervised learning approaches [15–16]. In weakly supervised learning, the labeling can be done on the WSI level, or even tumor level instead of pixel-wise annotations. This approach facilitates data preparation since there is no need for laborious pixel-wise annotations. For detection of BCC among other cancer types Campanella et al. [17] trained a weakly supervised convolutional neural network (CNN) model that demonstrated high accuracy. Their findings suggested that by integrating their model into clinical settings, around 75% of the slides could be excluded from the workload of pathology laboratories.

Further challenges in computational pathology include the large whole slide images (WSI). The main advantage of WSI is the high resolution, combined with the amount of context being displayed within the slide. Due to this ultra-high, giga-pixel dimension format-resolution of WSIs, which can reach up to 100,000 × 100,000 pixels (or higher) a set of unique challenges arise. Primarily, this exceptional level of detail necessitates the use of sophisticated software solutions capable of handling and efficiently rendering these large-scale images. This poses a substantial computational challenge, often solved by tiling the WSIs into smaller patches for individual analysis [18–19]. In cancer research, multiple-instance learning (MIL) has been a beneficial technique. MIL refers to the patches of the WSI as ”bags”, and if at least one patch contains tumor, the bag as a whole is labeled as tumor [20]. However, this approach overlooks the crucial correlations with neighboring patches. Graph convolutional networks (GCN) address this limitation by conceptualizing images as graph representations with nodes and edges. In WSIs the nodes are often represented by its patches, and their relationship as edges. This method has surpassed MIL in effectively considering spatial correlation and tissue morphology [21].

Inspired by the recent success of transformer architectures in natural language processing, Dosovitskiy et al. [22] adapted this approach to computer vision by introducing Vision Transformers (ViT). Instead of tokens, ViT uses patches of images as input. These patches are encoded with positional embeddings to preserve spatial information before being processed by a transformer with self-attention layers, allowing each patch to interact with others. This mechanism captures long-range dependencies between patches, integrating a global context across the entire image [22]. ViTs have since demonstrated remarkable results in histopathological image classification for various cancer types [23]. We have previously shown that a weakly supervised method using a combination of ViT together with a GCN graph transformer model can accurately detect and grade BCCs on excision specimens [18].

Pretrained models in pathology leverage machine learning algorithms trained on extensive and diverse digital histopathology datasets. These models capture complex representations of pathological features, enabling downstream tasks with improved accuracy and efficiency, even on limited datasets. These models can then be fine-tuned to perform specific tasks including disease classification, segmentation of ROI, etc. This approach is usually referred to as “transfer learning” when pre-trained models are adapted to new domains or specific tasks. By providing a pre-trained starting point that can leverage already learned patterns in similar tasks, a substantial amount of money, resources, and time can be saved [24].

Previous studies in digital pathology have highlighted key challenges in developing scalable and generalizable AI models. For instance, SISH (Self-Supervised Image Search for Histology) tackles scalability issues in WSI retrieval but struggles with domain generalization when faced with inter-site variability in staining and imaging protocols [25]. Similarly, PDLS (Pathology Deep Learning System) demonstrates robust performance on uncurated multi-site datasets but relies on extensive fine-tuning and confidence thresholds to manage inter-site variability and data artifacts [26]. Unlike these methods, our approach combines transfer learning, Vision Transformers (ViTs), and Graph Neural Networks (GNNs) to capture both localized morphological details and global contextual information. By fine-tuning on punch biopsies, our method bridges the gap between computational scalability and domain generalization. Hence, we aimed to fine-tune an existing weakly supervised machine learning model, originally trained on BCC excisions, to classify BCC in preoperative punch biopsies, utilizing transfer learning.

## Methods

This study is a retrospective, descriptive observational study at the Department of Pathology at Sahlgrenska University Hospital. The study was approved by the Swedish Ethical Review Authority (Dnr 2023-03774-01).

### Datasets

A total of 514 WSIs were retrospectively collected at the Department of Pathology at Sahlgrenska University Hospital retrospectively from years 2019 to 2024. Of the 514 WSIs, 261 WSIs represented BCCs and 253 tumor-free skin as shown in Fig S1. Only one WSI per patient was included. The tumor-free samples included a variety of dermatological conditions including scar fibrosis, reactive changes and common dermatoses (eczema, psoriasis, lichenoid inflammation). The slides were grouped into five classes (comprising four BCC subtypes and no-tumor). The data comprised 73 low-aggressive nodular tumors, 72 low-aggressive superficial tumors, 86 medium-aggressive tumors, 30 highly aggressive tumors, and 253 tumor-free samples, Table S1. For WSI scanning Hamamatsu NanoZoomer S360 scanner (Hamamatsu Photonics K.K., Shizuoka, Japan) at 40x mode (0.23 μm/pixel, 20x objective lens) was used. The scanned WSIs were added to the *Basal Cell Carcinoma Classification BCCC datase*t, Table S2 [27] originally used for pretraining the model.

Each file was weakly annotated at the WSI level, where the entire slide was assigned a single label corresponding to one of the four tumor aggressivity grades or as no-tumor. “Weakly” here refers to the absence of pixel-level annotations; instead, the annotation process labeled the entire WSI based on its overall characteristics, ensuring consistent labeling for use in the machine learning workflow. Slides with unsatisfactory quality were rescanned, resulting in the exclusion of one punch biopsy BCC slide which still after rescanning showed blurry areas covering most of the biopsy.

Each annotated WSI was assessed by two dermatopathologists (a junior specialist with two years of experience and a senior consultant with 9 years of experience). In instances of disagreement between the two (approximately 15 WSIs, ~ 5.7% of the BCCs), a third dermatopathologist (senior consultant, 15 years of experience) was consulted to establish a consensus agreement on the tumor grading. These annotations served as ground truth. The WSIs were randomly distributed among a training set (70%), a validation set (15%), and a test set (15%). To ensure balanced and proportional class representation across training, validation, and testing sets, we used Scikit-learn’s KFold method with fivefold cross-validation. This approach preserves the original class distribution within each fold, reducing the risk of overfitting and maintaining consistent class proportions throughout all phases. The final model was an ensemble, consolidating the predictions from each of the five folds.

The material were used in three classification tasks: first to distinguish between no-tumor and tumor (two classes, task 1), secondly between no-tumor, low-risk and high-risk subtype according to WHO grading system (three classes, task 2), and thirdly between no-tumor, superficial low, nodular low, medium-aggressive and high-aggressive according to Sabbatsberg’s grading system (five classes, task 3). In task 2, superficial and nodular BCCs are graded as ”low-risk”, while medium-aggressive and high-aggressive subtypes are graded as ”high-risk”.

Furthermore, to assess generalizability, an open-source dataset from Radboud University Medical Center (the Classification of Basal cell carcinoma, Risky skin tumors and Abnormalities, COBRA dataset, [28–29]) was used. From this external dataset of 3,588 punch biopsy WSIs, we included 1,794 WSIs containing BCC and 1,794 without BCC. The external dataset did not come with a 5-class division; however, it was divided into 3 classes: 0 - No tumor, 1 - Low-risk tumor, and 2 - High-risk tumor (Table S3). The internal and external test sets were evaluated separately.

### Model architecture and training

**Fig. 1:**
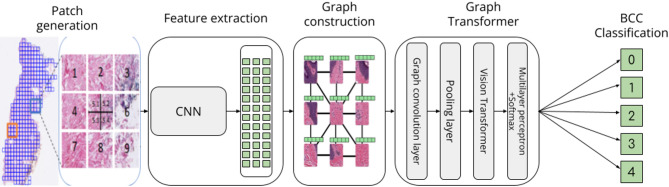
Flow chart of the key ideas in the model. The WSI is first divided into patches, and for each patch, a feature vector is generated using SimCLR. Graphs are then constructed to incorporate spatial information into each feature vector. These graphs are passed through the graph-transformer, which utilizes max pooling to reduce dimensionality before processing in the vision transformer

The main structure of the model can be seen in Fig. 1, in which the WSIs were divided into smaller patches using the open-source Python library OpenSlide, enabling analysis at multiple magnifications. This approach mirrors how a pathologist might zoom in to examine fine details within a specific region and then zoom out to understand the broader context of surrounding tissue. The choice of magnification for tiling affects the model’s predictive performance. High magnification provides detailed information but often lacks broader contextual insights, whereas low magnification tends to lose granular details. Through trial-and-error, a magnification level of 10X was selected as it captures both localized details and larger structural patterns in each patch. Patches containing less than 15% tissue were discarded.

The Simple Framework for Contrastive Learning (SimCLR) is a method of contrastive self-supervised learning that processes images to generate high-dimensional representational vectors without requiring labeled datasets for downstream tasks. Once the patches were created and stored, features for each patch were extracted using the SimCLR framework. The SimCLR framework, with ResNet18 as backbone, operates by augmenting an image through random transformations into pairs of the original image. This augmentation consists of cropping followed by resizing, adding color distortion, and applying random Gaussian blur. Augmented images form positive pairs if they originate from the same original image [30]. The resulting feature vectors, each containing 512 normalized numerical values, were stored as.csv files. These vectors were collected for each WSI, forming a feature matrix.

GCNs were utilized to capture among the patches by representing them as graphs. The GCN treated images as graphs, where nodes represented patches in the image and edges encoded the spatial relationships between patches, forming a grid of neighboring patches. This approach captures localized features in WSIs, such as tumor boundaries and tissue patterns, which depend on precise spatial arrangements. GCN aggregate features based on neighborhood information that led to higher model predictive performance [21].

The 512-length feature vectors representing each patch were used as nodes to create the adjacency matrix of the entire WSI. Connections were formed between edges and corners of the patches, with each patch having a maximum of 8 adjacencies. To reduce the graph’s dimensionality while preserving positional embeddings, a max-pooling layer was applied. A GCN block then aggregated information from neighboring nodes, reducing the feature vectors of each patch to 128 dimensions. Several distinct graphs were formed due to multiple unconnected tissue samples in each WSI, which were subsequently processed in the vision transformer.

The graph embeddings were fed to a vision transformer consisting of six transformer blocks. Each block comprised multi-head self-attention followed by a multi-layer perceptron. The final output underwent normalization, followed by a multi-layer perceptron and softmax to produce probability distributions for classification. In all classification tasks, the presence of a positive case or a specific subclass in one or more patches of a WSI determines the classification of the entire WSI. This approach effectively identifies the existence of a tumor or a specific tumor subclass based on the detection within individual patches.

The model was fine-tuned using a dataset split into training (70%), validation (15%), and test sets (15%). The validation set was used for early stopping to prevent overfitting, while cross-validation was employed to achieve model robustness across folds. The SimCLR feature extraction layer was kept frozen, while the GCN and transformer parameters were fine-tuned. The Adam optimizer was employed for learning rate updates, and the best models were saved based on validation accuracy. The training was performed for multiple classes, and the dataset was restructured for additional tasks, resulting in varying class divisions, (Fig S1). for classification.

## Results

First the non-fine-tuned pre-trained model’s inference on BCCC dataset was used as a benchmark to compare against the fine-tuned model. In the pretrained model, both the simCLR layer and the graph transformer had been trained on excisions of BCC as opposed to punch biopsies. Balanced accuracy was calculated using the balanced_accuracy_score function from the scikit-learn library. This metric was crucial due to the dominance of the “no tumor” class in the dataset, as traditional accuracy metrics could be misleading in such imbalanced cases. Balanced accuracy addresses this imbalance by equally weighting each class’s performance, ensuring a more accurate evaluation of the model’s performance across all classes.

Receiver Operating Characteristic (ROC) curve were used as graphical tools to illustrate the tradeoff between sensitivity and specificity across different threshold settings in the classifier evaluation. Different thresholds were systematically varied to compute the series of True Positive Rates and False Positive Rates for the model. By generating an ROC curve, we calculated the Area Under the Curve (AUC) value, which serves as a metric to assess the model’s ability to distinguish between different classes.

Additionally, a confusion matrix was constructed to visualize the classification outcomes. The results across the folds were aggregated and averaged to present the mean performance. The fine-tuned models were then compared to the pre-trained models using these metrics to evaluate improvements in classification performance.

### Classification on BCC dataset

The results for 2, 3, and 5 classes with a comparison between the non-fine-tuned pre-trained model and the fine-tuned models are presented in Table 1. The results show a consistent increase in accuracy, sensitivity, and specificity across all subclasses and tasks. This indicates that the fine-tuning of punch biopsy tissue has been successful in increasing performance in classification.

For the 2-class task, the sensitivity shows an increase from 62.6 to 89.5% for the No tumor class, and 79.5–95.0% for the Tumor class, which suggests that the model is better at detecting tumors. In the 3-class task, the sensitivity improvement is evident but varies by class. Specifically, the sensitivity for the “No tumor” class increases significantly from 60.5 to 89.5%, similar to the improvement observed in the 2-class model. However, it is notable that the gain in the High-risk class is less significant (68.9–72.2% respectively). This pattern also follows in the 5-class task, in which the sensitivity is increased across all classes, most notable in the No tumor class (27.8%), Type IA (30.9%), Type IB (40.0%), and Type II (29.2%) classes. Nonetheless, showing a smaller increase in the Type III class (20.0%) The specificity is also showing an increase across all tasks and sub-classes, meaning that the model is better at identifying negatives across all subclasses.

**Fig. 2:**
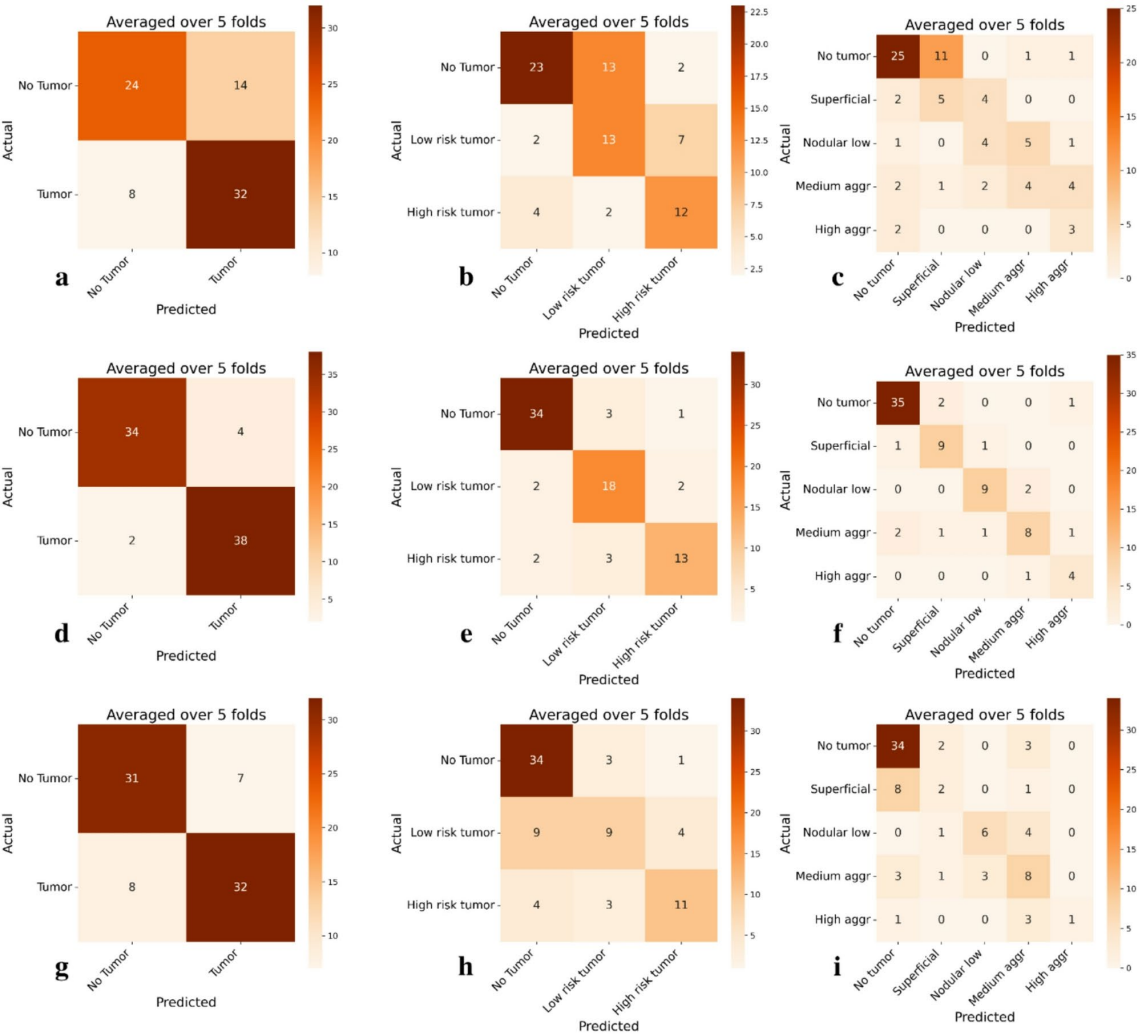
Confusion matrices for the pretrained and finetuned models across different classes in the BCC test set, first row showing the pretrained model and second row the finetuned. **a** 2-class pretrained model; **b** 3-class pretrained model; **c** 5-class pretrained model; **d** 2-class finetuned model; **e** 3-class finetuned model; **f** 5-class finetuned model; **g** 2-class model trained from scratch; **h** 3-class model trained from scratch; **i** 5-class model trained from scratch

As also shown confusion matrixes, the internal BCCC test-set results show better predictions after fine-tuning, Fig. 2.

**Fig. 3:**
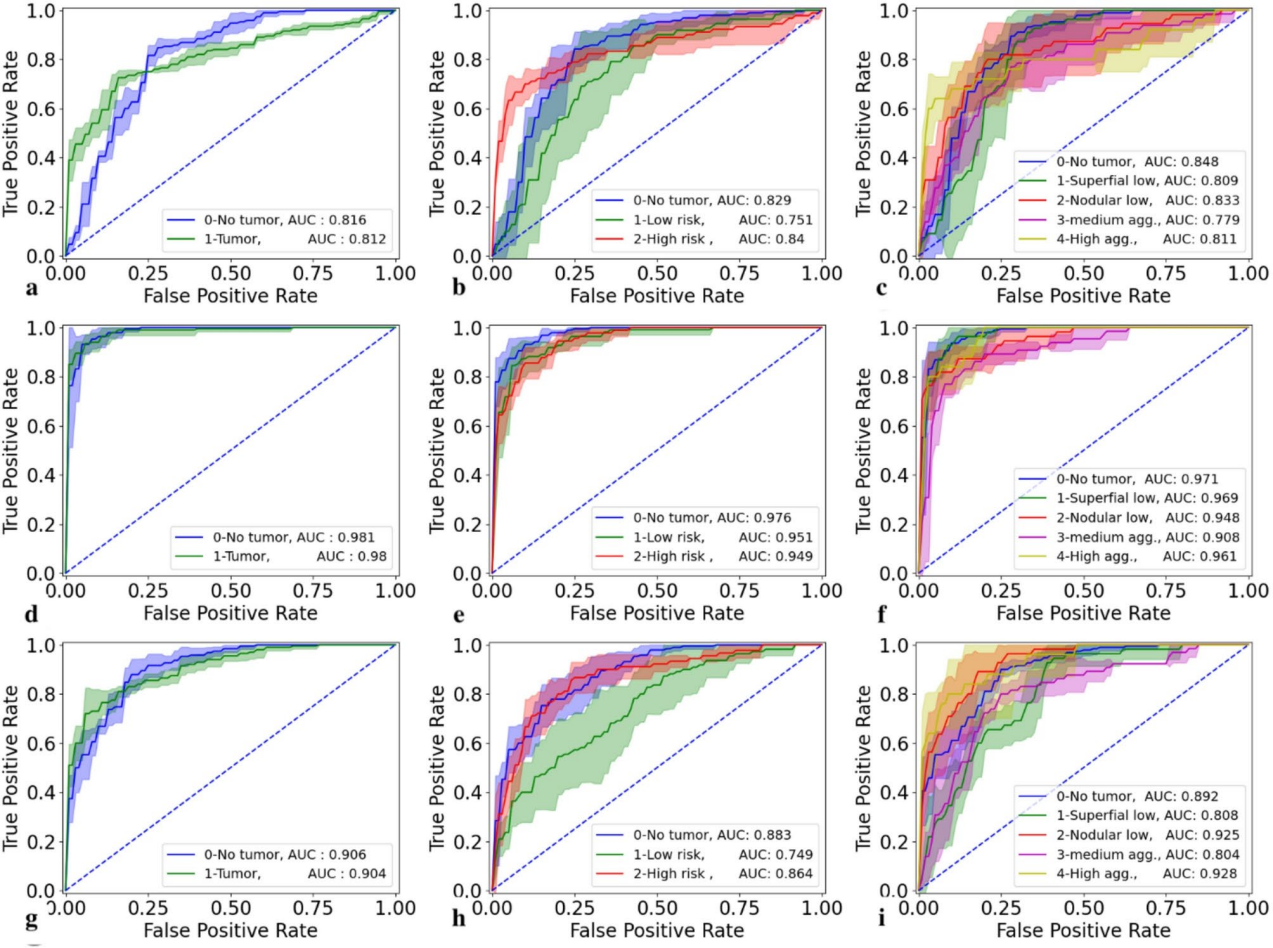
ROC curves for the pretrained and finetuned models across different classes for the BCCC test set. **a** 2-class pretrained model; **b** 3-class pretrained model; **c** 5-class pretrained model; **d** 2-class pretrained model; **e** 3-class pretrained model; **f** 5-class pretrained model; **g** 2-class model trained from scratch; **h** 3-class model trained from scratch; **i** 5-class model trained from scratch

As can be seen from Fig. 3 the fine-tuned model has higher AUC values across all tasks and subclasses, mirroring the results found in Table 1.

**Table 1:** Results for the BCCC dataset, the ablation study refers to the model which was trained from scratched and tested on the BCCC dataset

Task BCCC dataset	Sub-class	Balanced accuracy (%)			Sensitivity (%)			Specificity (%)		
		Pretrained	Finetuned	Ablation study	Pretrained	Finetuned	Ablation study	Pretrained	Finetuned	Ablation study
2 Classes	0 - No Tumor	71.1	**91.7**	**81.0**	62.6	**89.5**	81.6	79.5	**95.0**	80.5
	1 - Tumor				79.5	**95.0**	80.5	62.6	**89.5**	81.6
3 Classes	0 - No tumor	63.1	**82.1**	**69.0**	60.5	**89.5**	**90.0**	85.0	**88.6**	**67.0**
	1 - Low risk				60.0	**81.8**	**41.8**	73.9	**88.7**	**89.3**
	2 - High Risk				68.9	**72.2**	**57.8**	85.3	**94.5**	**91.7**
5 Classes	0 - No Tumor	50.0	**75.3**	**63.6**	64.7	**92.5**	**86.3**	84.0	**90.3**	**73.0**
	1 - Type IB				49.1	**80.0**	**21.8**	82.1	**95.0**	**94.0**
	2 - Type IA				40.0	**80.0**	**58.2**	91.0	**96.6**	**95.5**
	3 - Type II				32.3	**61.5**	**53.8**	91.4	**95.0**	**83.7**
	4 - Type III				60.0	**80.0**	**20.0**	91.5	**96.8**	**100**

### Classification on COBRA dataset

Table 2 shows that for the COBRA dataset, the balanced average accuracy increased after fine-tuning, in both tasks, indicating an overall increase in the classification performance. However, it is notable that the sensitivity decreases across the tumor classes, indicating that the model was slightly inferior in detecting the true positive tumors.

**Fig. 4:**
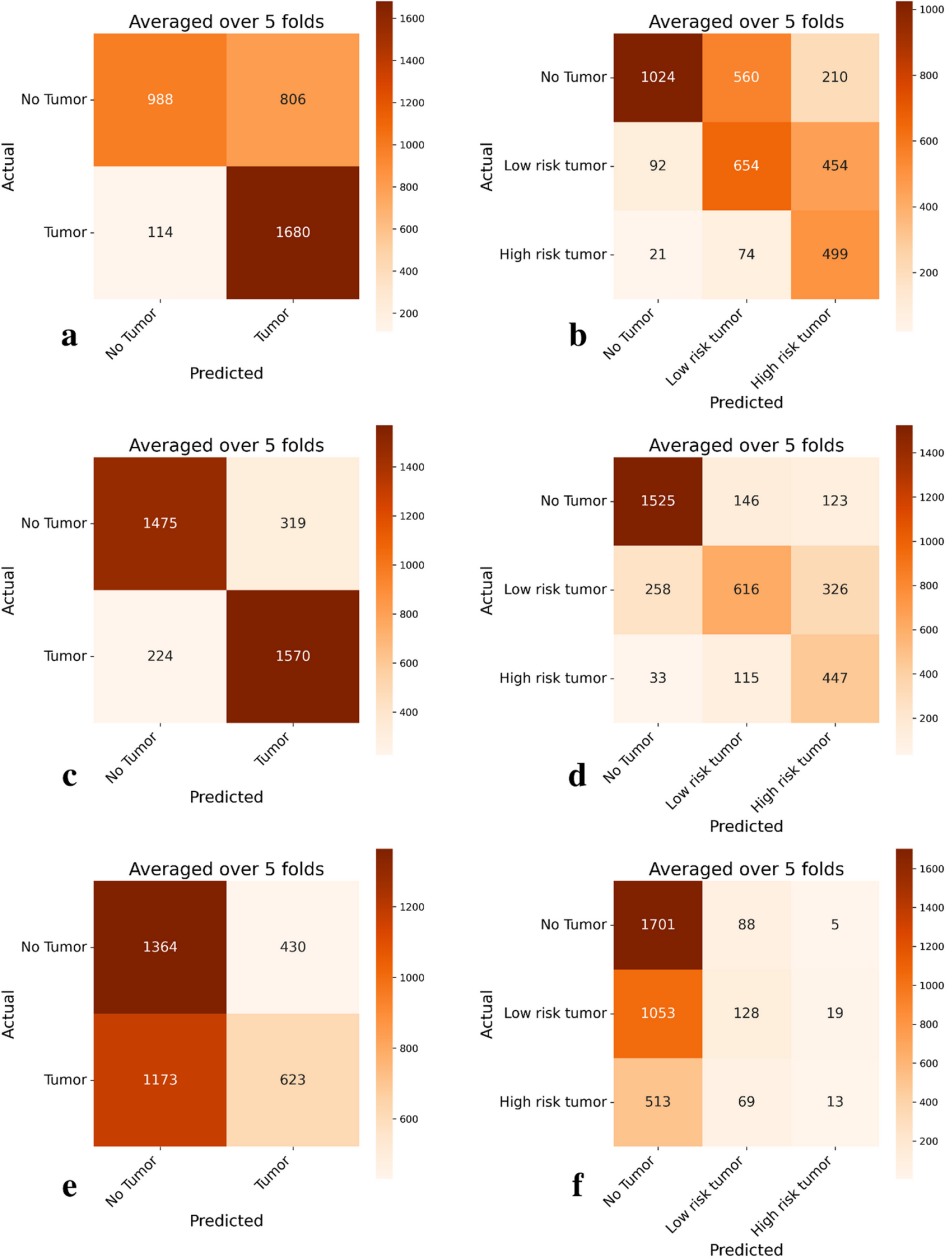
Confusion matrices for the pretrained on excisional BCC, and finetuned models across different classes in the COBRA test set. **a** 2-class pretrained model; **b** 3-class pretrained model; **c** 2-class finetuned model; **d** 3-class finetuned model; **e** 2-class model trained from scratch **f** 3-class model trained from scratch

In the external COBRA test set, the model shows signs of moving the predictions from the upper triangle of the confusion matrix towards the bottom triangle, for instance, in the 3-class task low-risk tumors predicted as No tumors increased from 92 to 258, while No tumors predicted as low tumors, decrease from 560 to 146. This pattern follows in the other classes as well, Fig. 4.

**Fig. 5:**
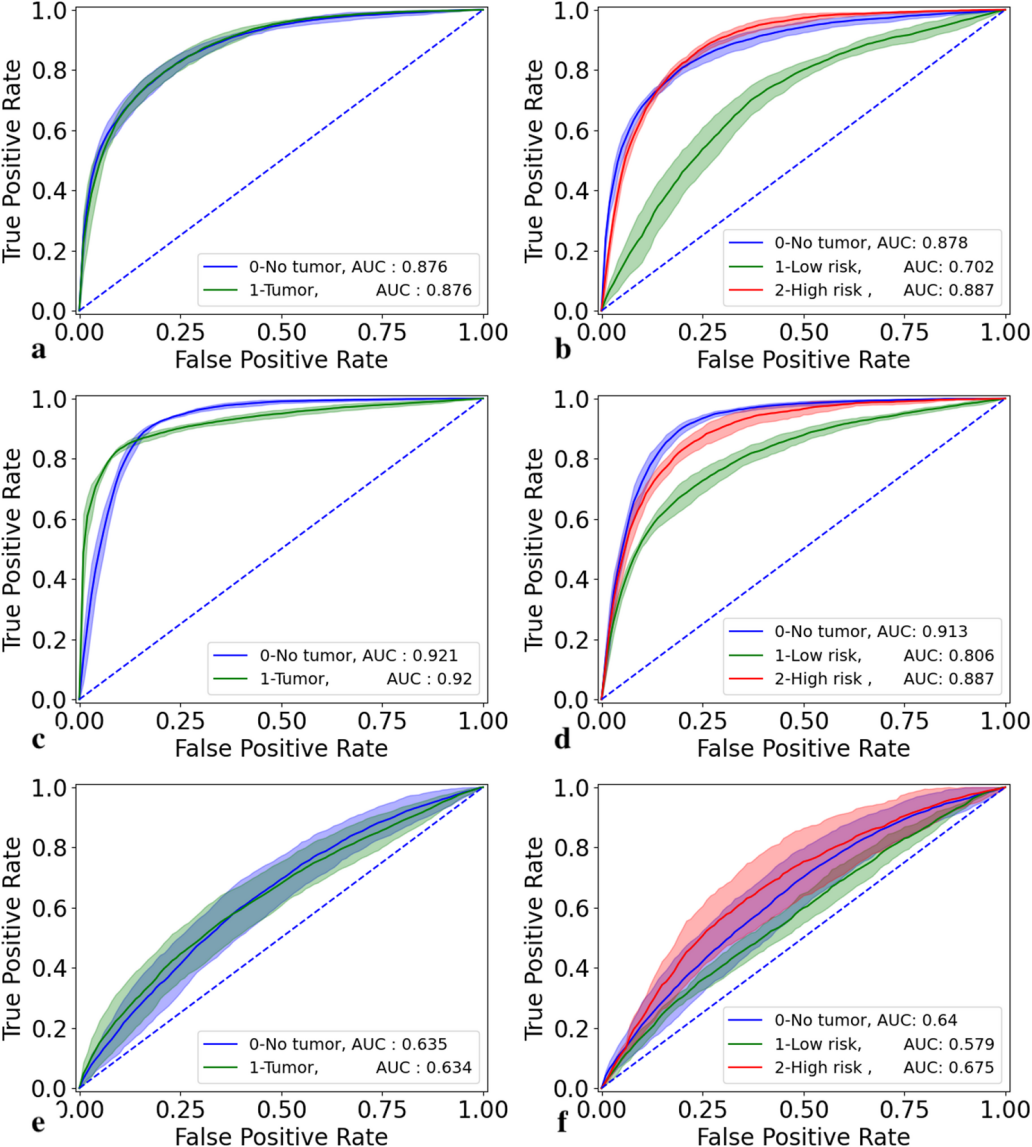
ROC curves for the pretrained and finetuned models across different classes for the external COBRA test set. **a** 2-class pretrained model; **b** 3-class pretrained model; **c** 2-class finetuned model and **d** 3-class finetuned model; **e** 2-class model trained from scratch **f** 3-class model trained from scratch

The results in the ROC curves in Fig. 5 also show a slight increase in the ability to distinguish between the different classes, especially in the two-class classification task after fine tuning.

**Table 2:** Results for the COBRA test set, the ablation study refers to the model which was trained from scratched and tested on the COBRA dataset

Task COBRA test set	Sub-class	Balanced accuracy (%)			Sensitivity (%)			Specificity (%)		
		Pretrained	Finetuned	Ablation study	Pretrained	Finetuned	Ablation study	Pretrained	Finetuned	Ablation study
2 Classes	0 - No Tumor	74.4	**84.9**	55.3	55.1	**82.2**	76.1	93.6	**87.5**	34.7
	1 - Tumor				93.6	**87.5**	34.7	55.1	**82.2**	76.1
3 Classes	0 - No tumor	65.2	**70.5**	51.3	57.1	**85.0**	94.8	93.7	**83.8**	12.8
	1 - Low risk				54.5	**51.4**	10.7	73.4	**89.1**	93.4
	2 - High Risk				84.0	**75.2**	2.3	77.8	**85.0**	99.2

### Ablation study

**Fig. 6:**
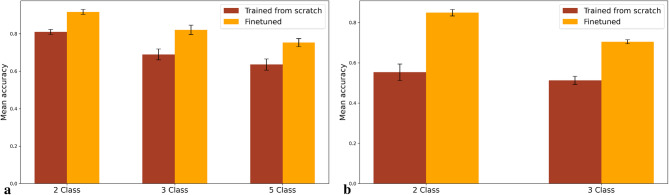
Comparison of performance between a model trained from scratch and the finetuned model. **a** show the increase in mean accuracy between the model trained from scratch and the finetuned model for the BCCC dataset, while **b** show the increase for the COBRA dataset

To demonstrate the benefits of utilizing a pre-trained SimCLR model on the excision BCC dataset, we conducted an ablation study. Specifically, we trained the SimCLR model using the BCC punch biopsy dataset and subsequently extracted embeddings from the trained model, which served as input for the graph transformer. The results, presented in Fig. 6, show that the pre-trained SimCLR model, fine-tuned on punch biopsy data, significantly outperforms the model trained from scratch. The mean accuracy increased from 81.0 to 91.7% for the 2-class classification task, from 69.0 to 82.1% for the 3-class classification task, and from 63.6 to 75.3% for the 5-class classification task. This improvement highlights the value of transfer learning. Furthermore, the model trained from scratch was also tested on the COBRA dataset. The results from the study showed that the model trained from scratch was unable to accurately capture the characteristics of the tumors on the COBRA dataset, achieving low mean accuracies, 55.3% and 51.3% as well as low sensitivity for all tumor classes.The detailed results are presented in Tables 1 and 2 as well as confusion and ROC-curves in Figs. 2, 3 and 4, and 5.

## Discussion

This study demonstrates significant improvement in classifying BCC in punch biopsies after fine-tuning a pretrained model. By freezing the whole slide image feature extractor (SimCLR model) and updating the weights in the Graph Transformer, the model’s accuracy, sensitivity, and specificity were enhanced across multiple tasks and subtypes. The fine-tuned model achieved accuracies of 91.7%, 82.1%, and 75.3%, and AUCs of 0.98, 0.95–0.98, and 0.91–0.97 for two, three, and five-class classifications, respectively. High performance was also observed on external COBRA dataset with accuracies of 84.9% and 70.5%, and AUCs of 0.92 and 0.81–0.91 for two and three-class classifications.

The original model, trained on excisional BCC biopsies, performed well on punch biopsies, but fine-tuning showed a total improvement of 20.6, 19.0, and 25.3% points in two, three, and five-class tasks, respectively. This resulted in 16, 15, and 20 additional samples being correctly classified on average. The model was originally trained with WSIs representing excisional tumor material where the whole tumor is excised with healthy skin margins. In contrast, in punch biopsies only a small partial central tumor area is represented. This difference can cause challenges for the AI model. Nonetheless, our work shows that fine-tuning could overcome this challenge.

The “no tumor” class included other skin abnormalities such as scar fibrosis and inflammatory dermatoses and in the external data set even epidermal dysplasia which could resemble a superficial BCC variant. Despite this, the model maintained high sensitivity and specificity across all tasks. The model’s performance decreased in three and five-class tasks, reflecting the increasing complexity of distinguishing between different subtypes, particularly aggressive subtypes characterized by small infiltrative tumor islands in a fibrotic background. Misclassified tumors often showed mixed growth patterns.

The external test set showed improved balanced accuracy, though not as prominently as the internal test set, likely due to differences in the scanning processes, staining protocols, and grading systems. Variations in these factors can introduce subtle visual differences that impact model performance, as the model may be less familiar with tissue characteristics shaped by alternative clinical protocols. The vast majority of the misclassified images in the COBRA dataset represented low aggressive superficial BCCs. In many of these images only minimal tumor foci was present. Further training is warranted in order to avoid this issue in future work.

Despite the imbalanced dataset, with certain BCC subtypes and the “no tumor” class dominating the samples, the fine-tuned model achieved high sensitivity and specificity across all classification tasks. This suggests that the model’s architecture and the transfer learning approach were robust enough to mitigate the effects of class imbalance without requiring specific adjustments, such as resampling or weighting.

The main enabler for deep learning in pathology is the accessibility of large amounts of labeled data with good quality. Currently, there is a shortage of high-quality, consistent datasets for training and testing machine learning models. When developing machine learning models for pathology purposes there are three main challenges; the complexity of the histopathology images making it difficult to obtain meaningful feature representations within the images, the limited number of labeled or annotated training data, and the size of the images subject to training [31]. These factors contribute to individual organizations and hospitals having a very limited capacity to obtain robust models for specific purposes. Firstly, it is time-consuming and can be hard to find the competence to label large data sets. It is also of course hard to obtain larger datasets due to patient data being sensitive for use and sharing between organizations.

Fine-tuning pre-trained models is beneficial for institutions with limited resources, as demonstrated by the significant improvements achieved with a smaller dataset. AI solutions in medical diagnostics can reduce pathology laboratory workloads. However, human oversight is necessary to validate AI-generated results, highlighting the need for careful integration of AI into clinical practice. This work focused on the most common skin cancer type, BCC causing heavy workload on pathology laboratories. Future work could involve training models on diverse datasets and including other tumor types to enhance generalizability and safety.

Our study’s strengths include using a weakly supervised approach and making our dataset publicly available for independent testing, which enhances the reproducibility and reliability of our findings. An additional strength in mitigating variability lies in our assessment of the model’s generalizability, wherein we evaluated its performance on an external dataset sourced from a distinct institution.

As the incidence of BCC is rapidly increasing, pathology laboratories are faced with an increasingly burdensome workload. Moreover, the grading process is susceptible to inter-observer variability. Consequently, the implementation of an automated diagnostic system may prove indispensable for future healthcare practices.

Moreover, the ablation study highlights the significant advantages of transfer learning through the use of a pre-trained SimCLR model. Fine-tuning the pre-trained model on the BCC punch biopsy data led to substantial improvements across all classification tasks, with mean accuracy increases from 81.0 to 91.7% for the 2-class task, 69.0–82.1% for the 3-class task, and 63.6–75.3% for the 5-class task. Similar results were found in the COBRA dataset, in which the increase in mean accuracy and sensitivity was even greater. These gains underscore the value of utilizing prior learned representations from excisional BCC data, which captured foundational patterns and structures relevant to BCC detection. In contrast, the model trained from scratch struggled to achieve similar levels of accuracy, likely due to the limited dataset size and complexity of the punch biopsy images. This suggests that transfer learning not only enhances model performance on small datasets but also supports robust feature extraction in complex histopathology tasks, where subtle distinctions, especially in BCC subtypes, are crucial. These findings reinforce the utility of pre-trained models in medical imaging and pathology, where access to large labeled datasets is often limited, and task-specific challenges require nuanced understanding of the visual data.

This study focused on a custom architecture capable of processing extremely large pathology images, where models like Vanilla and Swin Transformers are not directly applicable without significant redesign. While our primary goal was to demonstrate the value of domain-specific transfer learning, future work could explore scalable transformer adaptations, broader architectural benchmarking, and generalization across a wider range of skin pathologies to further validate and extend the approach.

## Conclusions

This work aimed to fine-tune an existing weakly supervised machine learning model, originally trained on BCC excisions, to classify BCC in preoperative punch biopsies, utilizing transfer learning. The results showed a significant increase in accuracy, sensitivity, and specificity across multiple classification tasks, with mean accuracies reaching 91.7%, 82.1%, and 75.3% for two-, three-, and five-class classification tasks, respectively, and corresponding AUCs up to 0.98. The fine-tuned model significantly outperformed the non-fine-tuned pretrained model and the model trained from the scratch. Mainly, this work underscores the potential of transfer learning and the leveraging of pre-trained models for fine-tuning toward specific tasks, which reduces the required training data significantly for smaller hospitals and institutes.

## Electronic supplementary material

Below is the link to the electronic supplementary material.

Supplementary Material 1

## Data Availability

The datasets generated and/or analysed during the current study are available at the BCCC dataset 10.23698/aida/bccc.

## Abbreviations

AUC: Area Under the Curve
BCC: Basal cell carcinoma
BCCC: Basal Cell Carcinoma Classification dataset
COBRA: Classification of Basal cell carcinoma, Risky skin tumors and Abnormalities dataset
CNN: Convolutional neural network
GCN: Graph convolutional networks
ML: Machine learning
MIL: Multiple-instance learning
ROC: Receiver Operating Characteristic
SimCLR: Simple Framework for Contrastive Learning
ViT: Vision Transformers
WSIs: Whole slide images
