# Supplementary material for: Enhancing basal cell carcinoma classification in preoperative biopsies via transfer learning with weakly supervised graph transformers
Source: BMC Med Imaging. 2025 May 16;25:166. doi: 10.1186/s12880-025-01710-4 (PMC12084905; doi:10.1186/s12880-025-01710-4)
Supplement: Supplementary file 1 — Supplementary Material 1 [file 12880_2025_1710_MOESM1_ESM.docx]

**Supplementary material**

***Datasets***

The BCCC dataset used for training and internal testing of the fine-tuned model is visualized in **Fig S1** and in **Table S1**. **Table S2** shows the original BCC excision dataset used for pretraining the model. The external COBRA test-set distribution is visualized in **Fig S2** and **Table S3**.


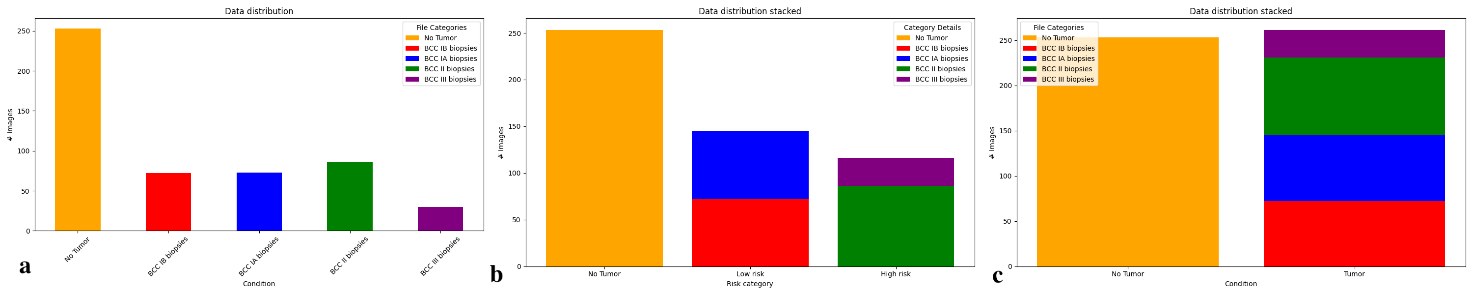


### **Figure S1:** Dataset distribution for BCCC biopsy dataset, showing the data used in the three tasks 5-class, 3-class, and 2-class classification. **a** Distribution of data across the 5 labels, **b** 3-class merge, **c** 2-class merge.

**Table S1:** Summary of the BCCC punch biopsy dataset used for fine-tuning.

| **Tumor Type** | **Count** |
| --- | --- |
| Low-aggressive nodular | 73 |
| Low-aggressive superficial | 72 |
| Medium-aggressive | 86 |
| Highly aggressive | 30 |
| No tumor | 253 |
| **Sum** | **514** |

**Table S2:** Summary of the BCCC excision dataset used for the pretraining the model.

|  | **All included** | | **Training and validation set** | | **Hold-out test set** | |
| --- | --- | --- | --- | --- | --- | --- |
|  | Number of cases | WSIs | Number of cases | WSIs | Number of cases | WSIs |
| **Total** | **479** | **1832** | **369** | **1435** | **110** | **397** |
| No tumor | 4 | 744 | 2 | 594 | 2 | 151 |
| Low aggressive superficial | 81 |  | 63 | 177 | 18 | 53 |
| Low aggressive nodular | 138 |  | 115 | 226 | 23 | 50 |
| Medium aggressive | 138 |  | 98 | 215 | 40 | 79 |
| High aggressive | 118 |  | 91 | 223 | 27 | 64 |

**Table S3:** Summary of the COBRA test set.

| **Tumor Type** | Count |
| --- | --- |
| No Tumor | 1794 |
| Low Risk Tumor | 1200 |
| High Risk Tumor | 594 |
| **Sum** | **3588** |


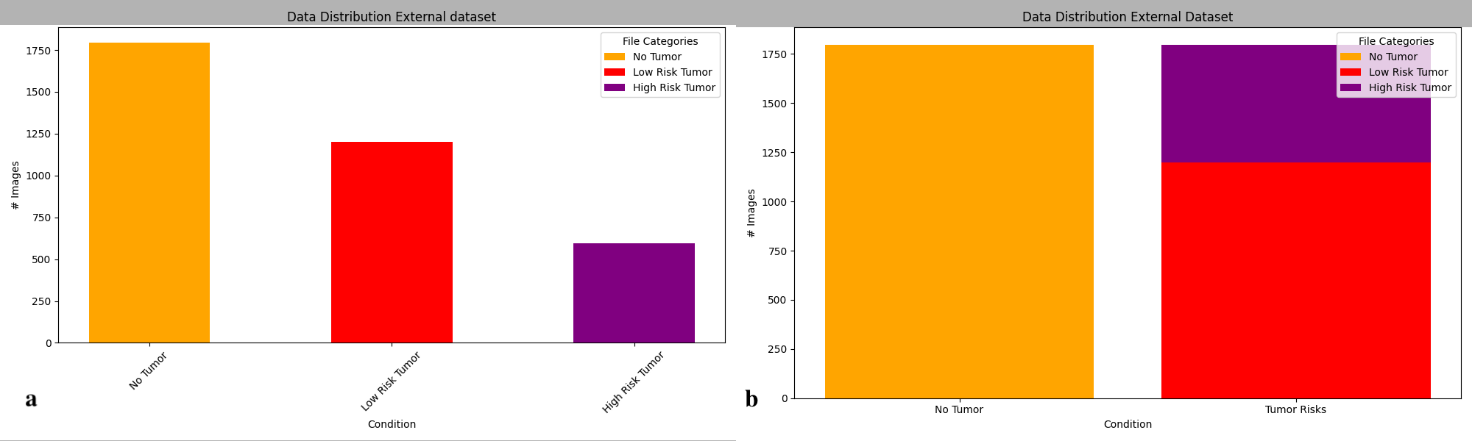


**Figure S2:** External dataset, showing the data used in the two tasks **a** 3class, and **b** 2-class classification
